# Dynamic Crosstalk Between TREM2^+^ Macrophages and the MASLD Hepatic Microenvironment

**DOI:** 10.3390/biomedicines14102167

**Published:** 2026-09-24

**Authors:** Xinting Li, Chuchu Yu, Aojie Mao, Yiran Peng, Yiyang Hu, Yu Zhao

**Affiliations:** 1 Key Laboratory of Liver and Kidney Diseases (Ministry of Education), Institute of Liver Diseases, Shuguang Hospital Affiliated to Shanghai University of Traditional Chinese Medicine, Shanghai 200021, China; 2 Shanghai Key Laboratory of Traditional Chinese Medicine, Shanghai 200021, China

**Keywords:** TREM2^+^ macrophages, metabolic dysfunction-associated steatotic liver disease (MASLD), metabolic dysfunction-associated steatohepatitis (MASH), hepatic fibrosis (HF), hepatocellular carcinoma (HCC), soluble TREM2 (sTREM2)

## Abstract

Chronic liver disorders, including metabolic/alcoholic steatohepatitis, hepatic fibrosis, and hepatocellular carcinoma, have complex pathogenesis in which triggering receptor expressed on myeloid cells 2 (TREM2) has an important role. In metabolic dysfunction-associated steatotic liver disease (MASLD), TREM2 is detectable in several hepatic non-parenchymal cell types, including Kupffer cells and hepatic stellate cells, but is most highly expressed in recruited monocyte-derived macrophages. TREM2 regulates macrophage lipid handling, inflammatory responses, and phagocytosis. TREM2^+^ macrophages are heterogeneous, and their functions vary with disease stage and local microenvironment. TREM2 has emerged as a therapeutic target in neurological diseases, while soluble TREM2 is being investigated as a noninvasive biomarker in MASLD. However, no TREM2-targeted therapy has yet been developed for MASLD. A clearer understanding of TREM2 signaling during progression from steatosis to metabolic dysfunction-associated steatohepatitis(MASH), hepatic fibrosis (HF), and hepatocellular carcinoma(HCC) may help identify new therapeutic strategies.

## 1. Introduction

The global prevalence of metabolic dysfunction-associated steatotic liver disease (MASLD) continues to rise and now affects approximately 30–40% of adults worldwide [1]. MASLD is also increasingly recognized in children and adolescents [2]. Its pathogenesis is best understood within a multiple-hit framework in which genetic susceptibility, insulin resistance, adipose-tissue dysfunction, hepatic lipotoxicity, mitochondrial and endoplasmic-reticulum stress, oxidative injury, gut–liver axis alterations, immune activation, and fibrogenic signaling interact over time [3]. These processes are closely linked: metabolic stress promotes hepatocyte injury and death, which in turn reshapes the hepatic immune and stromal microenvironment and drives progression from steatosis to metabolic dysfunction-associated steatohepatitis (MASH), hepatic fibrosis (HF), cirrhosis, and hepatocellular carcinoma(HCC) [4,5]. Although resmetirom and semaglutide have expanded treatment options for noncirrhotic MASH with moderate-to-advanced fibrosis, substantial residual disease burden and interpatient heterogeneity continue to support the need for additional mechanism-based therapies [6,7].

Macrophages, particularly recruited monocyte-derived macrophages (MDMs), are central components of this evolving microenvironment. During MASH progression, resident Kupffer-cell homeostasis is disrupted, and circulating monocytes enter the liver and remodel the macrophage pool. A prominent disease-associated population expresses triggering receptor expressed on myeloid cells 2 (TREM2), often together with CD9, GPNMB, APOE, and other lipid-handling genes. TREM2^+^ macrophages are heterogeneous and should not be viewed as uniformly beneficial or pathogenic; their functions depend on ontogeny, ligand availability, inflammatory tone, disease stage, and spatial niche. TREM2 is also detectable in other hepatic non-parenchymal cells, including subsets of Kupffer cells, dendritic cells, and hepatic stellate cells(HSCs) [8,9,10,11]. Although TREM2 modulation has been explored therapeutically in neurological disorders, its therapeutic relevance in MASLD remains to be established.

The spatial niche and surrounding tissue environment are major determinants of TREM2^+^ macrophage plasticity during inflammation, tissue repair, and regeneration [12,13,14]. This review therefore summarizes current evidence on the interplay between TREM2^+^ macrophages and the MASLD microenvironment, with emphasis on upstream regulation, stage-specific macrophage states, and downstream functions. We also discuss the potential clinical applications and limitations of targeting TREM2 in MASLD.

## 2. TREM2 Activating Ligands and Signaling Cascades

TREM2 is an immunoglobulin-superfamily receptor expressed predominantly in myeloid cells. The human *TREM2* gene is located at 6p21.1, whereas the murine homolog is located on chromosome 17 [15]. TREM2 contains a single extracellular V-type immunoglobulin domain, a short juxtamembrane region, one transmembrane helix, and a short cytoplasmic tail that lacks intrinsic signaling motifs [16,17]. Reported TREM2 ligands include fatty acids, low-density lipoprotein (LDL), apolipoproteins such as APOE, bacterial components, glycosaminoglycans, membrane phospholipids including phosphatidylserine and cardiolipin, and glycolipids such as sulfatides and cerebrosides [16,18,19]. α-secretases A Disintegrin and Metalloproteinase 10/17 (ADAM10/17) can cleave the TREM2 ectodomain near H157–S158, releasing soluble TREM2 (sTREM2). Subsequent γ-secretase-mediated cleavage of the membrane remnant terminates DNAX-activating protein of 12 kDa (DAP12)-associated signaling [16,20,21].

Because TREM2 lacks an intrinsic signaling domain, ligand binding is transmitted through associated adaptor proteins, mainly DAP12 and, in some settings, DNAX-activating protein of 10 kDa (DAP10). Src-family kinases phosphorylate the DAP12 immunoreceptor tyrosine-based activation motif (ITAM) and the DAP10 tyrosine–isoleucine–asparagine–methionine motif (YINM), thereby engaging downstream pathways that include the spleen tyrosine kinase–phosphatidylinositol 3-kinase–protein kinase B–mechanistic target of rapamycin (SYK–PI3K–AKT–mTOR), nuclear factor-κB (NF-κB), and mitogen-activated protein kinase (MAPK) signaling. These pathways regulate macrophage metabolism, survival, inflammatory responses, phagocytosis, and stress adaptation [16,22,23].

## 3. Defining TREM2^+^ Macrophages in MASLD

TREM2^+^ macrophages are highly plastic and heterogeneous across the course of MASLD. Their phenotypes differ according to spatial location, disease stage, cellular origin, and transcriptional program, and are continuously shaped by local microenvironmental cues [24,25].

Single-cell and spatial transcriptomic studies have refined the definition of TREM2^+^ macrophage populations in metabolic and fibrotic liver disease. Lipid-associated macrophages (LAMs), first described in obese adipose tissue, express high levels of TREM2, CD9, and genes involved in lipid uptake, lysosomal function, and lipid metabolism [26]. Similar TREM2^+^CD9^+^ LAM populations have since been identified in diseased liver. In MASLD, most LAMs arise from recruited monocyte-derived macrophages, although a subset of embryonically derived resident Kupffer cells can also acquire a LAM-like state in diseased mouse and human liver [11,26]. These cells preferentially accumulate in lipid-rich, injured regions, where TREM2-dependent recognition and clearance of dying cells can support tissue repair. Related LAM populations are also found in metabolically stressed white and brown adipose tissue [26,27]. NASH-associated macrophages (NAMs) similarly express TREM2, CD9, and GPNMB, together with transcriptional programs related to lipid metabolism, lysosomal activity, and cellular stress [25]. Unlike the original adipose-tissue LAM population, NAMs were defined in diseased liver and increase with NASH severity [25] (Table 1).

Scar-associated macrophages (SAMs) are a TREM2^+^CD9^+^ macrophage population enriched in fibrotic liver niches. Lineage-tracing studies indicate that they arise predominantly from recruited bone marrow-derived macrophages rather than resident Kupffer cells. SAMs localize near activated hepatic stellate cells and extracellular-matrix-rich scar regions, where they participate in macrophage–stromal communication and matrix remodeling [25,26] (Table 1).

*TREM2* is also highly expressed by subsets of tumor-associated macrophages (TAMs) in HCC. TAMs derive from both resident Kupffer cells and recruited monocyte-derived macrophages and undergo extensive transcriptional reprogramming within the tumor microenvironment [28,29,30]. TREM2-high TAM populations are generally associated with immunosuppressive macrophage programs and an immunosuppressive tumor microenvironment (Table 1).

LAMs, NAMs, SAMs, and TREM2^+^ TAMs share overlapping core transcriptional features but represent context-dependent manifestations of a broader TREM2^+^ disease-associated macrophage program. Their phenotypic diversity reflects differences in cellular origin, disease stage, and the metabolic, inflammatory, fibrotic, or neoplastic niche.

**Table 1:** Major TREM2^+^ macrophage states across MASLD progression.

Macrophage Type	Predominant Tissue Distribution	Representative Highly Expressed Genes	Major Functional Features	References
LAMs	Steatotic, inflammatory, and fibrotic hepatic niches; also white and brown adipose tissue	*TREM2*, *CD9*, *GPNMB*, *LIPA*, *LPL*, *CTSB*, *CTSL*, *FABP4/5*, *LGALS1/3*, *CD36;* human-enriched *APOC1*, *TIMP1/3*	Lipid handling, lysosomal catabolism, efferocytosis, and tissue repair; functions vary with disease stage and niche	[11,26,31]
NAMs	Steatotic and inflammatory regions of diseased MASH liver	*TREM2*, *CD9*, *GPNMB*, *MS4A7*, *CTSD*, *H2-AB1*	Disease-associated lipid handling and efferocytosis; *MS4A7*-high states can support NLRP3 inflammasome activation	[32,33]
SAMs	Fibrotic and collagen-rich scar regions	*TREM2*, *CD9*, *SPP1*, *GPNMB*, *FABP5*, *CD63*, *LGALS3*, *CCR2*, *TNFSF12*	Scar-niche communication and matrix remodeling; may exhibit either restorative/MMP-rich or profibrogenic SPP1/LGALS3-rich programs	[25,26,34,35]
TAMs	HCC tumor microenvironment and invasive niches	*TREM2*, *SPP1*, *APOE*, *PD-L1*, *LGALS3*, *CD68*, *CD163*, *CCR2*, *CXCL8*	Immunosuppression, Treg recruitment, CD8^+^ T-cell exclusion, metabolic adaptation, and tumor support	[30,36,37,38]

Lipid-associated macrophages (LAMs), NASH-associated macrophages (NAMs), scar-associated macrophages (SAMs), tumor-associated macrophages (TAMs).

## 4. Upstream Regulation of TREM2^+^ Macrophages in MASLD

TREM2 expression is closely linked to tissue context and disease stage. In dynamic MASLD models, TREM2 expression is relatively low during simple steatosis but rises as hepatocyte injury, MASH, and fibrosis develop [39]. MASLD progression is accompanied by dysregulated lipid metabolism, insulin resistance, lipotoxicity, oxidative and endoplasmic-reticulum stress, hepatocellular injury, and increased intestinal permeability [40]. Among these processes, lipotoxicity, hepatocyte injury, immune activation, and fibrogenic signaling have the strongest evidence for promoting the emergence, expansion, or localization of TREM2^+^ macrophages. Other factors, including insulin resistance, mitochondrial dysfunction, oxidative stress, ER stress, and the gut–liver axis, are more likely to influence TREM2^+^ macrophage states indirectly by remodeling the hepatic microenvironment rather than through a single TREM2-specific pathway.

### 4.1. Recruitment of TREM2^+^ Macrophage Precursors

In the steady-state liver, resident yolk-sac-derived Kupffer cells (KCs) typically express only low levels of TREM2, while TREM2^+^ macrophages that massively expand during MASH progression originate mainly from peripherally recruited monocytes. Among relevant pathways, the C-C motif chemokine ligand2–C-C motif chemokine receptor 2 (CCL2–CCR2) axis is one of the best-characterized pathways supplying these precursors. Hepatocyte injury and inflammatory signaling increase hepatic CCL2, which recruits CCR2^+^ monocytes that can pass through a CCR2^high^/C-X3-C motif chemokine ligand 1(Cx3cl1)^high^ transitional state. After entering the liver, these cells are reprogrammed by lipid-rich, inflammatory, and fibrotic niche signals and gradually acquire TREM2^+^CD9^+^ glycoprotein non-metastatic B (GPNMB)^+^ LAM features; a subset can also differentiate toward monocyte-derived Kupffer cells. *Ccr2* deletion or pharmacological CCR2 inhibition reduces hepatic *Trem2* abundance largely by limiting monocyte recruitment rather than by directly suppressing Trem2 transcription [11,24,41]. A related mechanism operates in HCC, where MCP-1 (CCL2) produced by peritumoral hepatocytes promotes recruitment of monocyte-derived macrophages into tumor tissue [42]. The CCL15–CCR1 axis also contributes to recruitment of immunosuppressive myeloid cells in HCC. Integrated single-cell analyses of NASH-to-HCC progression found enrichment of CCR1 in TREM2^+^ macrophages together with increased tumor-derived CCL15 during malignant transformation, supporting preferential recruitment and positioning of TREM2^+^ immunosuppressive macrophages. In addition, malignant hepatocyte-derived midkine (MDK) can recruit and localize TREM2^+^ macrophages through nucleolin (NCL) [43]. Overall, these pathways primarily regulate the supply and positioning of macrophage precursors; acquisition of the TREM2^+^ state is subsequently shaped by the local tissue niche (Table 2).

### 4.2. Transcriptional and Epigenetic Reprogramming

#### 4.2.1. LXR-Dependent Transcriptional Reprogramming

Liver X receptor-α (LXR-α) is predominantly expressed in macrophages, hepatocytes and adipose tissues [44]. In MASH hepatic macrophages, LXR upregulation not only enhances cholesterol outflow but also elevates TREM2 expression [45]. In vitro, LXR agonists increase *TREM2* expression in THP-1-derived macrophages and promote a lipid-associated macrophage-like program [38]. The vacuolar ATPase subunit *Atp6v0d2* is also linked to this pathway. In MASLD/MASH samples and CDAHFD-fed mice, macrophage *Atp6v0d2* expression correlates with *Trem2* and *Cd9*. In *Atp6v0d2*-deficient BMDMs, the LXR agonist T0901317 restores *Trem2* expression, reduces proinflammatory polarization, and improves cholesterol efflux and efferocytosis of apoptotic hepatocytes [45].

The transcriptional output of LXR is strongly context-dependent. In NASH, widespread enhancer remodeling in Kupffer cells redirects LXR occupancy toward disease-associated regulatory regions. AP-1 family factors, including (activating transcription factor 3) ATF3, are increased under NASH conditions and co-localize with LXR at regulatory regions near *Trem2* and *Cd9*, and recruit the histone acetyltransferase (HAT) p300. p300 catalyzes acetylation of histone H3 lysine 27 (H3K27). These sites show increased chromatin accessibility and H3K27ac, consistent with enhanced activity of disease-associated enhancers [24,46,47]. Thus, LXR should not be viewed simply as a direct inducer of TREM2; rather, its transcriptional effects depend on the enhancer landscape established by the local disease environment.

#### 4.2.2. Additional Transcriptional Regulators: HIF1A and PPARγ

Hypoxia-inducible factor 1α (HIF1A) has also been implicated in LXR-associated regulation of TREM2. In hypoxic HCC lesions, macrophage *HIF1A* expression increases and is associated with a shift toward TREM2^+^ macrophage states [42]. In THP-1 cells, LXR agonists increase *HIF1A* and downstream glycolytic and hypoxia-responsive genes, including *GLUT1*, *HK2*, and *VEGFA*. *HIF1A* knockdown substantially reduces LXR agonist-induced *TREM2* expression, supporting a role for *HIF1A* in this response [38] (Figure 1).

In addition to LXR, TREM2 expression also correlates positively with PPARγ and Bax. In the CCl_4_-induced mouse model of hepatic fibrosis and cirrhosis, peroxisome proliferator-activated receptor γ (PPARγ) associated pathway activity is suppressed, whereas transcripts including *Spp1*, *Trem2*, and *Mmp7* are upregulated, and their expression levels positively correlate with fibrosis severity [48]. In contrast, transcriptomic analysis of myeloid-specific Trem2-knockout macrophages showed increased *Bax* and reduced *PPARγ* expression [49]. Adelmidrol, an antifibrotic agent that increases PPARγ activity, reduces scar-associated *Trem2*^+^ macrophages, and this effect is attenuated by the PPARγ inhibitor GW9662 [50]. These findings support an association between PPARγ signaling and TREM2^+^ macrophage states but do not establish a single linear PPARγ–BCL2-associated X protein (Bax)–TREM2 pathway.

### 4.3. Hepatocyte-Derived Lipids, Lipoproteins, and Damage-Associated TREM2 Ligands

TREM2 can recognize a broad range of lipid and lipoprotein-associated signals. In early MASLD, macrophages are exposed mainly to excess lipids and lipoprotein cargo. As disease progresses to MASH, hepatocyte injury adds apoptotic-cell phospholipids, extracellular lipid droplets, oxidized lipids, and cellular debris to this environment. Fibrosis introduces a persistent scar-associated niche in which stromal and inflammatory signals further reshape TREM2^+^ macrophage states. In HCC, tumor-cell metabolic reprogramming can generate additional lipid signals, including oxidized LDL (oxLDL), that favor immunosuppressive TREM2^+^ TAM states. Thus, both ligand availability and the cellular context of TREM2 engagement change across disease stages.

#### 4.3.1. Sphingosine-1-Phosphate (S1P)

Hepatocyte injury during MASH releases multiple damage-associated signals, including sphingosine-1-phosphate (S1P). Current evidence supports two distinct links between S1P and TREM2. First, S1P can act through macrophage S1PR1 to increase *Trem2* mRNA and protein expression and enhance phagocytic capacity [51,52,53,54]. Second, S1P can bind directly to TREM2 and promote TREM2-dependent phagocytosis independently of classical S1P receptors. Microscale thermophoresis, surface plasmon resonance, and LC–MS/MS studies identified residue D104 as important for this interaction [54]. S1P receptor modulation can also alter the composition of hepatic macrophage populations. In FFC-fed MASH mice, Etrasimod and Amiselimod reduced inflammatory macrophage accumulation and lowered *Mcp1*, TNF-α, and IL-1β expression, while *Trem2* transcripts increased together with changes in the CD11b^+^ macrophage compartment [55]. These findings suggest that changes in hepatic *Trem2* after S1P-receptor modulation may partly reflect redistribution of macrophage subsets rather than direct induction alone (Table 3).

#### 4.3.2. Lipid Droplets

Damaged steatotic hepatocytes can release extracellular lipid droplets (LDs). Spatially, TREM2^+^CD9^+^GPNMB^+^ lipid-associated macrophages preferentially accumulate within hepatic crown-like structures surrounding lipid-laden, injured hepatocytes [24,39]. Experimental studies also support a role for hepatocyte-derived LDs in shaping the hepatic macrophage compartment. LDs isolated from steatotic livers of high-fat-diet-fed mice are taken up by both Kupffer cells and monocyte-derived macrophages after intravenous administration, promoting a TREM2^+^ NAM program. In mice fed a MASH diet for 3 months, repeated LD administration for 4 days further increased ALT and AST and upregulated *Trem2*, other NAM-associated genes, and proinflammatory genes [56]. Full-length membrane TREM2 has also been identified as a receptor involved in macrophage uptake of triglyceride-rich lipoproteins (TRLs), supporting a broader role for TREM2 in lipid handling [57]. However, the molecular mechanism by which extracellular LDs induce Trem2 expression remains unclear. Moreover, no study has directly compared defined short- and long-duration LD exposure within the same experimental system. A strict time-dependent switch in LD-mediated regulation of TREM2 expression and macrophage function therefore remains to be established (Table 3).

#### 4.3.3. Phosphatidylserine (PS) and Phosphatidylethanolamine (PE)

During the transition from steatosis to MASH, lipotoxic hepatocyte apoptosis increases exposure of aminophospholipids such as phosphatidylserine (PS) and phosphatidylethanolamine (PE) on dying cells. In intact Neuro2a cell membranes, PS and PE are mainly located on the inner leaflet; during apoptosis they become exposed on the cell surface and activate TREM2–DAP12 signaling in a dose-dependent manner. Physical separation of apoptotic cells from reporter cells largely abolishes this response, supporting a requirement for contact-dependent ligands. Purified PS and PE also activate TREM2 signaling, whereas phosphatidylcholine does not [58]. Although these experiments were performed outside the liver, they provide a plausible mechanism by which apoptotic hepatocytes could engage TREM2-expressing macrophages in MASH. Direct validation in hepatic macrophages is still needed (Table 3).

#### 4.3.4. APOE and APOE-Containing Lipoproteins

APOE is produced abundantly in the liver and is a major apolipoprotein in chylomicron remnants, very-low-density lipoprotein (VLDL) remnants, intermediate-density lipoprotein (IDL), and a subset of high-density lipoprotein (HDL) particles [59,60]. It supports hepatic clearance of remnant lipoproteins through receptors including receptors including low-density lipoprotein receptor (LDLR) and low-density lipoprotein receptor-related protein 1 (LRP1). APOE is also one of the best-characterized TREM2 ligands, although most direct binding studies were performed in neuroimmune systems. Independent studies in 2015 demonstrated specific APOE–TREM2 binding [61,62]. All three common APOE isoforms can bind TREM2, but reported differences in affinity are modest and not fully consistent across experimental systems. Later work suggested that non-lipidated APOE can bind human TREM2 and that lipidation is not strictly required [63]. TREM2 also recognizes other apolipoproteins and lipoprotein particles, including clusterin (CLU)/APOJ, APOA, APOB, and LDL, and can facilitate uptake of LDL, APOE, and CLU [64]. APOE serves not only as a direct TREM2 ligand but also as an integral component of the lipid-handling transcriptional program in TREM2^+^ macrophages. Following efferocytosis of apoptotic neurons, microglia undergo transition toward a disease-associated phenotype, accompanied by enhanced APOE signaling and activation of disease-related transcriptional programming [65]. These findings are relevant to MASH because hepatic TREM2^+^ LAMs express *Apoe*, *Gpnmb*, and *Cd9* and are strongly engaged in lipid handling and phagocytosis. Nevertheless, the relative importance of free APOE, APOE-containing particles, and specific lipidated forms in the MASLD liver remains unresolved (Table 3).

#### 4.3.5. Oxidized Lipid

TREM2 can also recognize oxidized phospholipids. In Alzheimer’s disease models, the TREM2 R47H variant shows reduced binding to PS, oxidized PS, and PE compared with wild-type TREM2, supporting recognition of oxidized phospholipid species [66]. In atherosclerosis, *Trem2* deletion reduces oxLDL uptake by foam macrophages and impairs cholesterol efflux, proliferation, and survival [67], although this does not by itself establish oxLDL as a direct biochemical ligand. In HCC, SQLE-high tumor cells generate an oxidized lipid-rich environment in which oxLDL induces *Trem2* and *Spp1* expression and activates SYK-dependent signaling in macrophages. Genetic or pharmacological inhibition of TREM2, as well as SYK inhibition, reduces this response, supporting an oxLDL–TREM2–SYK–CEBPα pathway that favors TREM2^+^ secreted phosphoprotein 1 (SPP1)^+^ TAM formation [37]. Oxidative stress and lipid peroxidation also increase during MASH, but the extent to which oxidized lipids directly activate hepatic LAMs through TREM2 remains uncertain (Table 3).

### 4.4. Inflammatory Signaling and Post-Translational Control of TREM2

Chronic inflammation is a major feature of MASH and affects both macrophage recruitment and TREM2 function. Persistent lipotoxic injury increases inflammatory cytokines and chemokines such as CCL2, promoting recruitment of CCR2^+^ monocytes into the liver. Once recruited, these cells can differentiate into TREM2^+^ disease-associated macrophages in response to the lipid-rich and damage-associated niche. ADAM17 is a major sheddase of human TREM2 and cleaves membrane-bound TREM2 at H157–S158, releasing sTREM2 and reducing full-length cell-surface TREM2 [68]. Membrane-spanning 4-domains subfamily A member 7 (MS4A7) is enriched in TREM2^+^ NAMs and directly interacts with NOD-like receptor family pyrin domain containing 3 (NLRP3) to facilitate inflammasome activation and IL-1β release; *Ms4a7* deficiency attenuates LD-triggered inflammation and MASH severity [32]. However, current evidence does not establish a direct regulatory interaction between MS4A7 and TREM2. In parallel, uptake of extracellular LDs can induce an anti-inflammatory response that depends in part on intact TREM2 signaling. In wild-type macrophages, LD uptake suppresses inflammatory signaling and NLRP3 activation, whereas this effect is blunted in the absence of TREM2 [56]. Thus, proinflammatory MS4A7–NLRP3 activity and protective TREM2-dependent lipid-handling responses can coexist within TREM2^+^ NAMs, and TREM2 expression should not be equated with uniformly active TREM2 signaling. During prolonged hypernutrition, TNF-α and IL-1β enhance ADAM17-mediated TREM2 shedding. This can shift the balance toward loss of functional membrane TREM2 and impair efferocytosis of dying hepatocytes, contributing to a feed-forward cycle of inflammation [53].

### 4.5. Fibrotic Niche-Dependent Reprogramming

TREM2^+^CD9^+^ macrophages expand in cirrhotic liver and accumulate within collagen-rich scar niches, where they are commonly referred to as SAMs [34,35]. Collagen itself has not been established as a direct TREM2 ligand; instead, dying cells, lipid debris, and other signals within the fibrotic niche are more likely to contribute to TREM2 activation [39]. These macrophages also engage in extensive crosstalk with activated HSCs and scar-associated mesenchymal cells [34].

Transforming growth factor-beta-induced protein (TGFBI) has recently emerged as one extracellular-matrix-associated signal linking the fibrotic niche to TREM2^+^ macrophage differentiation. In fibrotic liver, TGFBI is increased in nonparenchymal cells, including HSCs and macrophages. It promotes macrophage proliferation, migration, and differentiation toward TREM2^+^CD9^+^ profibrotic states through integrin αvβ3–AKT–ERK signaling and increases platelet-derived growth factor subunit B (PDGF-B) expression. TGFBI also enhances platelet-derived growth factor receptor beta (PDGFRβ) expression in HSCs through integrin αvβ3–focal adhesion kinase (FAK)–signal transducer and activator of transcription 3 (STAT3) signaling, supporting a positive feedback loop within the fibrotic niche. These findings position TGFBI as a niche-derived regulator of TREM2^+^CD9^+^ macrophage expansion rather than as a direct TREM2 ligand or transcriptional regulator [69].

TGF-β also contributes to the formation and maintenance of TREM2^+^CD9^+^ scar-associated macrophages. In both CDA-HFD and GAN diet models, myeloid-specific *Tgfbr1* deletion reduces the hepatic Trem2^+^ macrophage population and worsens liver injury, inflammation, and fibrosis, indicating that myeloid TGF-β signaling helps maintain this compartment [70]. In vitro, GM-CSF, IL-17A, and TGF-β1 can drive human monocytes toward macrophages expressing scar-associated markers [71]. TGF-β1 activates Small Mothers Against Decapentaplegic family member 3 (SMAD3), and this response is restrained by neural precursor cell expressed developmentally down-regulated 4-like (NEDD4L), an E3 ubiquitin ligase that promotes degradation of phosphorylated SMAD family member 3 (SMAD3). Loss of *Nedd4l* prolongs SMAD3 activation and increases the ability of macrophages to activate HSCs [72]. However, direct transcriptional regulation of Trem2 by TGF-β/SMAD3 or related fibrotic-niche signals has not been firmly established.

### 4.6. Gut–Liver Axis as an Indirect Regulator of TREM2^+^ Macrophage States

Altered gut microbiota and microbial metabolites are repeatedly associated with MASLD severity. One direct link to TREM2 comes from evidence that TREM2 can recognize negatively charged bacterial surface structures. TREM2-expressing cells bind both *E. coli* and *Staphylococcus aureus*, and TREM2-Fc binding to *E. coli* can be inhibited by LPS, lipoteichoic acid, peptidoglycan, and other anionic molecules [73]. Whether portal microbial products directly engage TREM2 on hepatic macrophages during MASLD/MASH, however, remains uncertain. More broadly, intestinal dysbiosis, impaired barrier integrity, and altered microbial metabolism influence hepatic lipid metabolism, innate immune activation, monocyte recruitment, and fibrogenic signaling [74]. The gut–liver axis is therefore likely to shape TREM2^+^ macrophage states mainly by altering the hepatic metabolic and inflammatory environment (Table 3).

**Table 3:** Experimentally validated and putative TREM2 ligands, lipid cargoes, and upstream regulators relevant to metabolic liver disease.

TREM2 Ligand/Regulator	Evidence Status	Effects on TREM2^+^ Macrophages	Main Experimental Evidence	References
S1P	Directly validated ligand	Directly binds TREM2 and activates TREM2-dependent phagocytic signaling. In MASH, S1P can also increase *Trem2* expression through S1PR1 signaling.	Molecular docking, MST, SPR, LC–MS/MS, TREM2–DAP12-transfected CHO cells, and TREM2 knockdown in microglia	[54]
Extracellular LDs	Functionally implicated; direct binding to TREM2 remains unproven	Promote monocyte recruitment and the emergence of TREM2^+^ NAMs. Following LD uptake, intact TREM2 signaling contributes to suppression of inflammatory signaling and NLRP3 inflammasome activation.	LD uptake/efferocytosis models, Trem2-deficient BMDMs, immunofluorescence, and in vivo LD administration	[32,56]
TRLs	Directly validated receptor cargo/ligand	Full-length macrophage TREM2 facilitates TRL uptake. TREM2 shedding impairs macrophage-mediated TRL clearance and favors accumulation of APOC3-rich TRLs.	Human cohorts with prediabetes and hepatic steatosis, mouse models, Trem2 deficiency, preservation of full-length TREM2, and TRL uptake assays	[57]
PS/PE	Directly validated signal-transducing ligands	Exposure of PS/PE on apoptotic cells activates TREM2–DAP12 signaling and promotes recognition and phagocytic clearance of apoptotic cells.	TREM2–DAP12 reporter assays, apoptotic Neuro2a cells, aminophospholipid masking, and purified PS/PE	[58]
APOE	Directly validated ligand	Binds TREM2 and facilitates uptake of APOE- and lipoprotein-associated cargo, thereby contributing to TREM2-dependent lipid handling.	TREM2-Fc pull-down, ELISA, biochemical interaction assays, and cellular uptake assays	[61,62,63,64,65]
CLU/APOJ	Directly validated ligand	Binds TREM2 and facilitates uptake of lipoprotein- or Aβ-associated cargo.	Protein microarray, biolayer interferometry, and cellular uptake assays	[61,62,63,64]
APOA-containing apolipoproteins	Binding supported, but less extensively validated than APOE or CLU/APOJ	May contribute to TREM2-mediated recognition of apolipoprotein- and lipoprotein-associated cargo.	Protein microarray and biochemical binding assays	[64]
APOB-containing lipoproteins	Binding supported	May participate in TREM2-mediated lipoprotein recognition and uptake.	Protein microarray and lipoprotein-binding assays	[64]
LDL	Directly supported lipoprotein ligand/cargo	TREM2 facilitates LDL uptake, whereas disease-associated TREM2 variants impair this function.	Protein microarray, biolayer interferometry, TREM2 overexpression, and *Trem2*-deficient microglia	[64]
oxPS	Direct binding supported	Wild-type TREM2 binds oxPS, whereas the R47H variant shows reduced lipid-binding capacity, suggesting recognition of oxidized phospholipid species by TREM2.	Lipid-binding assays, ELISA, and comparison of WT and R47H TREM2 ectodomains	[64]
oxLDL	Functionally validated TREM2-dependent stimulus; direct physical binding remains incompletely established	Activates the TREM2–SYK–CEBPα axis in HCC-associated macrophages and promotes the emergence of TREM2^+^SPP1^+^ tumor-associated macrophages.	BMDMs, THP-1 cells, *Trem2* knockout, TREM2 blockade, soluble TREM2 competition, SYK inhibition, and HCC models	[66]
Whole bacteria/bacterial anionic ligands	TREM2 binding and reporter activation demonstrated; precise molecular ligand identity remains incompletely defined	TREM2 recognizes negatively charged microbial surface components and can trigger DAP12-dependent signaling in reporter systems.	TREM2-Fc binding to Gram-positive and Gram-negative bacteria, reporter-cell activation, and competition with LPS, LTA, peptidoglycan, and dextran sulfate	[73]

Sphingosine-1-phosphate (S1P), lipid droplets (LDs), triglyceride-rich lipoproteins (TRLs), phosphatidylserine (PS), phosphatidylethanolamine (PE), apolipoprotein A/B/E (APOA/B/E), low-density lipoprotein (LDL), oxidized phosphatidylserine (oxPS), oxidized LDL (oxLDL).

## 5. Functional Consequences of TREM2^+^ Macrophages Across MASLD Progression

### 5.1. TREM2^+^ Macrophages Limit MASH Progression

#### 5.1.1. Regulation of Hepatic Lipid Metabolism and Inflammatory Homeostasis

TREM2^+^ macrophages expand during MASLD progression, and hepatic TREM2 expression generally increases with MASH severity [25]. In human MASLD/MASH samples and mouse models, TREM2 expression in intrahepatic macrophages correlates with genes involved in fatty-acid metabolism, inflammation, and fibrosis [49]. These associations, however, largely reflect expansion of TREM2^+^ macrophage states in response to tissue injury and should not be interpreted as evidence that TREM2 itself is pathogenic. Experimental loss-of-function studies instead show that intact myeloid TREM2 signaling can support metabolic adaptation and limit inflammatory injury. Deletion of *Trem2* in hematopoietic cells or macrophages impairs lipid handling and alters differentiation toward LAM- and SAM-like states, accompanied by increased expression of inflammatory and chemotactic genes [75]. *Trem2*-deficient mice on a high-fat diet and myeloid-specific Trem2-deficient mice on an AMLN diet also develop higher ALT/AST levels, greater hepatic triglyceride accumulation, more severe steatosis, and increased insulin resistance [49,76].

One important function of TREM2 is to help macrophages cope with excess lipid and lipoprotein cargo. In LPS-stimulated *Trem2-deficient* BMDMs, expression of *Lpl* and Cd36 is reduced, consistent with impaired lipid uptake and handling [77]. Global *Trem2* deletion during high-fat feeding is likewise associated with worsened insulin resistance, adipose inflammation, adipocyte hypertrophy, and cell death [76]. Evidence from disease-reversal models further supports a role for TREM2 in tissue recovery. Five weeks after vertical sleeve gastrectomy, hepatic TREM2^+^ LAMs show increased lysosomal activity, lower inflammatory signaling, and enhanced phagocytic capacity; these benefits are blunted in *Trem2*-deficient mice [78].

Myeloid TREM2 also limits macrophage pyroptosis through PI3K–AKT signaling. Compared with controls, myeloid-specific *Trem2* deletion reduces PI3K/AKT activity and increases NLRP3, cleaved gasdermin D (GSDMD), caspase-1 p20, and mature IL-1β, whereas TREM2 overexpression reverses these changes [49]. TREM2 also influences macrophage programs associated with inflammation resolution. Myeloid *Trem2* deficiency reduces hepatic and BMDM-derived TGF-β1, while TREM2-replete macrophages show stronger repair-associated signaling [49,71,79]. These findings help explain why TREM2 expression can increase in severe MASH even though loss of TREM2 worsens steatosis and inflammation. TREM2 may also affect hepatocyte mitochondrial homeostasis through macrophage–hepatocyte communication. Hou et al. reported that *Trem2*-deficient macrophages release exosomes enriched in miR-106b-5p, which suppresses hepatocyte mitofusin 2 (MFN2) and disrupts mitochondrial fusion and energy metabolism. Conversely, macrophage TREM2 overexpression improved hepatic energy metabolism in a model of NAFLD-associated sepsis [80] (Figure 2).

#### 5.1.2. Clearance of Dying Hepatocytes and Efferocytosis

Hepatocyte death is a key event in the transition from steatosis to sustained inflammation and fibrosis. Inadequate clearance of dying hepatocytes allows continued release of mitochondrial DAMPs, nucleic acids, lipid peroxides, and other inflammatory cellular contents. Macrophage efferocytosis therefore contributes not only to removal of dead cells but also to resolution of injury-induced inflammation and restoration of tissue homeostasis.

TREM2 contributes to the ability of hepatic macrophages to clear apoptotic hepatocytes. In vitro, *Trem2-*deficient macrophages show impaired uptake of apoptotic hepatocytes and stronger inflammatory responses after lipopolysaccharide(LPS) challenge. TREM2 signaling through PI3K/PDK1/AKT or SYK has been linked to changes in mTOR activity, cellular metabolism, survival, and phagocytic capacity [8,81]. CD14 can cooperate with TREM2 during recognition and clearance of dying cells [26,77,82]. In the absence of TREM2, impaired clearance of necrotic and apoptotic hepatocytes increases exposure to mitochondrial DAMPs, which further amplifies macrophage inflammatory signaling and worsens experimental fibrosis [83] (Figure 2).

### 5.2. Stage- and Cell-State-Dependent Effects of TREM2^+^ Macrophages on Hepatic Fibrosis

Available evidence indicates that the effects of TREM2 in liver fibrosis depend strongly on disease stage, cellular origin, and local microenvironment. In some settings, TREM2 supports repair by promoting dead-cell clearance, macrophage survival, and matrix remodeling. In others, TREM2^+^ scar-associated macrophages acquire *SPP1-* and *LGALS3*-rich programs and participate in profibrotic crosstalk with hepatic stellate cells.

Across several acute and chronic liver-injury models, De Ponti et al. showed that both recruited monocyte-derived LAMs and a subset of resident Kupffer cells that acquire a LAM-like state contribute to tissue repair. TREM2 expression in both populations is required for efficient clearance of dead cells; *Trem2* deletion delays injury resolution and worsens fibrosis [11]. TREM2^+^ macrophages may also contribute to matrix remodeling. Integrated single-cell analyses of mouse NASH, experimental cirrhosis, and human cirrhosis identified expansion of monocyte-derived TREM2^+^ macrophages with fibrosis progression and a strong association between TREM2 abundance and matrix metalloproteinase signatures [35]. These data support a matrix-remodeling function for at least a subset of TREM2^+^ SAMs.

TREM2^+^ macrophages can also adopt profibrotic states. In fibrotic and cirrhotic liver, TREM2^+^CD9^+^ SAM abundance correlates with fibrosis stage, and these cells express genes such as *Spp1*, *Lgals3*, and *Ccr2* while interacting closely with collagen-producing mesenchymal and endothelial cells [34]. TREM2^+^CD9^+^GPNMB^+^ LAMs also contribute to hepatic crown-like structures (hCLS), focal niches associated with inflammation and fibrosis [24,84]. In AMLN and CDAHFD models, myeloid *Trem2* deficiency worsens steatosis and inflammation but can reduce fibrosis and impair hCLS formation [49]. suggesting that TREM2 may help maintain macrophage states that support local matrix deposition under chronic MASH conditions. Separate studies have reported TREM2 expression in HSCs, where TREM2-dependent SYK–integrin subunit alpha V (ITGAV) signaling may promote HSC activation [85]; this HSC-intrinsic effect should be distinguished from macrophage TREM2. More recently, p21^+^TREM2^+^ senescent macrophages have been identified in aged and MASLD mouse liver and in human cirrhosis. DNA damage and persistent cholesterol loading can promote this state, and depletion of these cells reduces hepatic inflammation and steatosis in MASLD mice [86].

Taken together, TREM2 cannot be classified simply as either antifibrotic or profibrotic. During acute injury, early repair, or fibrosis regression, TREM2-dependent clearance of dying cells can support tissue recovery. During persistent MASH and active fibrosis, however, specific TREM2^+^ macrophage states may contribute to scar maintenance and matrix deposition through hCLS formation and macrophage–stromal crosstalk. In advanced chronic disease, TREM2^+^ macrophages may also acquire senescent or other dysfunctional states. Fibrotic outcome therefore depends not only on TREM2 abundance but also on macrophage origin, transcriptional state, spatial niche, and whether the liver is progressing toward or recovering from injury (Figure 2).

### 5.3. TREM2^+^ Macrophages Promote an Immunosuppressive HCC Microenvironment

Single-cell, spatial-omic, and immunohistochemical studies consistently identify TREM2 enrichment in a subset of LAM-like tumor-associated macrophages (TAMs) in HCC. These TREM2^+^ macrophages are enriched in tumor lesions, can arise from *S100A8^+^* monocytes, and are associated with greater macrophage infiltration and poorer prognosis [38]. TREM2^+^ TAMs are also increased in HCC with microvascular invasion and are linked to an immunosuppressive, pro-metastatic tumor niche [43].

TREM2^+^ TAMs can promote HCC progression by reshaping the T-cell microenvironment. TREM2^+^ LAM-like macrophages are associated with Treg accumulation and with cell–cell communication programs that favor immunosuppressive T-cell states [38]. Lipid-laden TREM2^+^PD-L1^+^ macrophages can secrete CCL20 and recruit CCR6^+^ Tregs [87]. TREM2^+^ TAMs may also contribute to immune exclusion. After TACE, these macrophages produce galectin-1, which increases endothelial PD-L1 and limits transendothelial migration of CD8^+^ T cells. *Trem2* deletion increases intratumoral CD8^+^ T-cell infiltration, reduces tumor growth, and improves response to anti-PD-L1 therapy in this setting [88].

Metabolic crosstalk between tumor cells and TREM2^+^ TAMs provides another mechanism linking these macrophages to HCC progression. In the tumor microenvironment, oxLDL generated in association with tumor-cell lipid metabolism activates TREM2/DAP12–SYK signaling and increases C/EBPα activity. C/EBPα can then promote *Trem2* and *Spp1* transcription, establishing a positive-feedback program that favors TREM2^+^SPP1^+^ TAMs [37]. These macrophages increase tumor-cell invasion, reduce sensitivity to effector cytokines such as IFN-γ and TNF, and suppress CD8^+^ T-cell activity. *Trem2*-deficient BMDMs and TAMs also show increased CXCL10, CCL5, TNF-α, and IL-1β; macrophage-derived IL-1β can in turn promote pyruvate kinase M2 (PKM2)-linked glycolysis in HCC cells [89]. Together, these findings illustrate that TREM2 can suppress selected inflammatory outputs while still supporting an immunosuppressive TAM state.

TREM2 function in HCC is strongly cell-type dependent. In contrast to the tumor-promoting effects associated with TREM2^+^ TAMs, TREM2 expressed by tumor cells or hepatic stellate cells has shown tumor-suppressive effects in some models. In HCC cells, TREM2 can interact with β-catenin and reduce AKT/glycogen synthase kinase 3 β (GSK3β) signaling, thereby limiting β-catenin accumulation and tumor-cell proliferation [90]. Consistent with this context dependence, whole-body *Trem2* deficiency can increase tumor burden in DEN-induced and fibrosis-associated HCC models, accompanied by greater liver injury, oxidative stress, inflammation, and compensatory regeneration. TREM2 overexpression in HSCs has also been reported to reduce Wnt ligand secretion and suppress Wnt/β-catenin signaling in HCC cells [9]. By contrast, other studies have reported antitumor effects of compounds that reduce TREM2 in HCC cells, including echinacoside [91]. These apparently divergent findings further emphasize the need to distinguish macrophage TREM2 from TREM2 expressed by other hepatic cell types.

Overall, TREM2 has cell-type- and context-dependent effects in HCC. TREM2^+^ TAMs are generally associated with immunosuppression and reduced response to immunotherapy. In contrast, TREM2 expressed by tumor cells or HSCs can exert tumor-suppressive effects in selected experimental models. These distinct roles should be considered separately when evaluating TREM2-directed therapies (Figure 2).

## 6. Clinical Translation of TREM2 in MASLD

### 6.1. Therapeutic Targeting of TREM2 and TREM2^+^ Macrophage States

TREM2 has entered clinical drug development, but most programs have focused on neurodegenerative diseases and cancer. No TREM2-targeted therapy is currently approved for MASLD, and direct TREM2-based interventions in this field remain preclinical. AL002, a TREM2 agonistic monoclonal antibody developed for Alzheimer’s disease, completed the randomized phase 2 INVOKE-2 trial [92]. Despite clear target engagement, the trial did not meet its primary clinical endpoint. This experience illustrates that increasing TREM2 activity alone may not be sufficient and supports greater attention to cell type, functional state, and disease stage when designing TREM2-directed therapies.

In MASLD/MASH, prolonged overnutrition and sustained inflammation can impair TREM2 function by increasing receptor shedding. TNF-α and IL-1β enhance ADAM17 activity, promoting cleavage of membrane-bound TREM2, reducing functional cell-surface receptor, and increasing sTREM2. Loss of membrane TREM2 can impair efferocytosis and contribute to persistent inflammation. Preserving membrane TREM2 may therefore be more relevant than simply increasing TREM2 transcription or systemically activating the receptor. However, systemic inhibition of ADAM10/17 would not be TREM2-selective because these proteases act on many substrates and have broad physiological functions. More selective approaches that preserve membrane TREM2 without globally suppressing ADAM10/17 may therefore offer a better therapeutic window.

This concept has been tested using a synthetic cleavage-resistant TREM2 (CRT). Dong et al. reconfigured the stalk and transmembrane regions of TREM2 to reduce proteolytic shedding while retaining TREM2–DAP12 signaling. CRT mRNA was delivered with macrophage-targeted lipid nanoparticles, generating CRT-expressing macrophages in vivo. This approach enhanced macrophage efferocytosis, reduced uncleared apoptotic cells, and improved inflammatory and disease phenotypes in mouse models of MASH and atherosclerosis [93]. These findings support selective preservation of functional membrane TREM2 in defined macrophage populations rather than systemic TREM2 agonism or nonspecific ADAM10/17 inhibition. Important questions remain, however. It is not yet clear whether CRT signaling is regulated by physiological ligand availability to the same extent as endogenous TREM2, or what the long-term consequences of sustained membrane TREM2 expression might be. Potential effects on macrophage persistence, fibrogenic programs, immunosuppression, tissue specificity, and systemic immune homeostasis will need to be assessed.

Nanodelivery strategies have also been explored to improve spatial selectivity of TREM2-directed therapy in tumors. For example, carbon-nanotube-based systems have been used to co-deliver PD-L1 blockade and TREM2-targeted nucleic acid cargo, enabling local checkpoint inhibition and TREM2 silencing within tumor lesions [94]. Although these approaches can enhance T-cell activity and suppress tumor growth in preclinical models, their tissue specificity, long-term safety, and scalability remain to be established.

Modulating the upstream metabolic and immunoregulatory signals that shape TREM2^+^ macrophage states may provide another therapeutic approach. In HCC, SQLE-dependent cholesterol dysregulation increases oxLDL and promotes TREM2^+^SPP1^+^ TAM formation through TREM2–SYK–C/EBPα signaling. Pharmacological SQLE inhibition reduces this immunosuppressive macrophage population and improves CD8^+^ T-cell responses to anti-PD-1 therapy [37]. A separate study in NASH-driven hepatocarcinogenesis showed that myeloid Trem2 deficiency enhances neutrophil extracellular trap (NET) clearance and reduces NET-associated TLR4 signaling, CXCR4 expression, and Treg accumulation, thereby improving antitumor immunity and response to checkpoint blockade [95]. This mechanism has therefore been demonstrated specifically in NASH-associated HCC and should not be generalized to all HCC models. Reprogramming TREM2^+^ TAMs may also improve cellular immunotherapy. GPC3-targeted CAR-T cells can directly recognize HCC cells, but their efficacy is limited by the immunosuppressive tumor microenvironment [96]. *Trem2* deficiency can shift TAMs from an SPP1-high immunosuppressive state toward a CXCL9-high antitumor state, accompanied by increased CD40 expression, fatty-acid oxidation, and CXCL9 production. CD40 agonism partially reproduces this macrophage reprogramming and enhances CD8^+^ T-cell and CAR-T-cell recruitment [97]. These findings suggest that clinically tractable pathways such as CD40 may provide an alternative means of redirecting TREM2^+^ macrophage states without directly blocking TREM2.

Overall, therapeutic strategies should focus less on changing total TREM2 abundance and more on preserving beneficial TREM2 functions or suppressing pathogenic TREM2^+^ macrophage programs in a disease-stage- and niche-specific manner.

### 6.2. sTREM2 as a Noninvasive Biomarker and Translational Readout

Soluble TREM2 (sTREM2) is generated mainly by proteolytic cleavage of the extracellular domain of membrane-bound TREM2. Circulating sTREM2 may therefore reflect both expansion of TREM2^+^ macrophages and turnover of membrane TREM2. Current clinical evidence supports sTREM2 primarily as a biomarker of the macrophage response associated with MASH. Hendrikx et al. reported in mouse and human studies that circulating sTREM2 increases with NASH severity and parallels recruitment and expansion of hepatic TREM2^+^ monocyte-derived macrophages [75,98]. In cohorts enriched for individuals at high risk of MASLD by transient elastography, plasma sTREM2 was nearly twofold higher in patients with histologically confirmed NASH. The AUROC for NASH diagnosis was 0.83 in an independent validation cohort and 0.92 in an exploratory cohort. Proposed cutoffs of ≤38 ng/mL and ≥65 ng/mL provided higher sensitivity for rule-out and higher specificity for rule-in, respectively [99]. sTREM2 correlated more strongly with steatosis, ballooning, and lobular inflammation than with fibrosis stage, suggesting that it may reflect active steatohepatitis more closely than cumulative scar burden. Kothari et al. similarly reported higher plasma sTREM2 in biopsy-confirmed NASH, with an AUROC of approximately 0.81 for distinguishing NASH from simple steatosis [100]. Other cohorts, including studies in women with MASLD, have also linked circulating sTREM2, hepatic TREM2 abundance, and macrophage infiltration with histological disease severity.

sTREM2 may also have value for longitudinal monitoring. In a MASLD cohort with serial liver biopsies, Wernberg et al. found that sTREM2 decreased in parallel with improvement in NAS. A panel combining sTREM2, PRO-C3, and HOMA-IR identified histological improvement with an AUROC above 0.75, whereas FIB-4 and the NAFLD fibrosis score were less sensitive to changes in disease activity [101]. These findings support further evaluation of sTREM2 as a pharmacodynamic marker in clinical trials. In patients undergoing liver resection, higher preoperative sTREM2 has also been associated with advanced fibrosis, post-hepatectomy liver failure, and poorer survival; the AUROC for post-hepatectomy liver failure was 0.708 [102]. These observations extend the potential clinical relevance of sTREM2 but do not establish it as a stand-alone diagnostic or surrogate endpoint.

## 7. Strengths, Limitations, and Future Perspectives

A strength of this review is its integration of macrophage ontogeny, ligand biology, transcriptional and epigenetic regulation, inflammatory signaling, fibrotic-niche remodeling, and the gut–liver axis within a stage-dependent framework for TREM2^+^ macrophage biology. Several limitations should also be noted. This is a narrative rather than a systematic review, and much of the mechanistic evidence discussed here comes from mouse models, BMDMs, microglia, or other non-hepatic systems. Direct validation in human hepatic macrophages remains limited. In addition, the field is evolving rapidly, and some mechanistic and translational conclusions may need to be revised as new evidence emerges.

Important limitations also remain in the current evidence base. TREM2 expression, TREM2^+^ macrophage abundance, membrane receptor signaling, and sTREM2 release represent distinct biological processes but are not always clearly separated in published studies. The dominant hepatic TREM2 ligands and the mechanisms that drive transitions between protective and pathogenic macrophage states are also incompletely defined. Most mechanistic evidence still comes from experimental models, while longitudinal, spatially resolved, and causal studies in human MASLD remain scarce. These gaps should be addressed before TREM2-directed therapies can be translated confidently into clinical practice.

Overall, the metabolic, inflammatory, fibrotic, and neoplastic microenvironments that emerge during MASLD progression shape the heterogeneity of TREM2^+^ macrophages and influence their recruitment, TREM2 expression, and function. Future work should define the direct interactions between TREM2^+^ macrophages and the hepatic microenvironment, determine whether ligands and regulatory mechanisms identified in non-hepatic systems also operate in MASLD, particularly through longitudinal validation in human cohorts, and develop strategies that modulate specific TREM2^+^ macrophage states within appropriate stage-specific therapeutic windows.

## Figures and Tables

**Figure 1:**
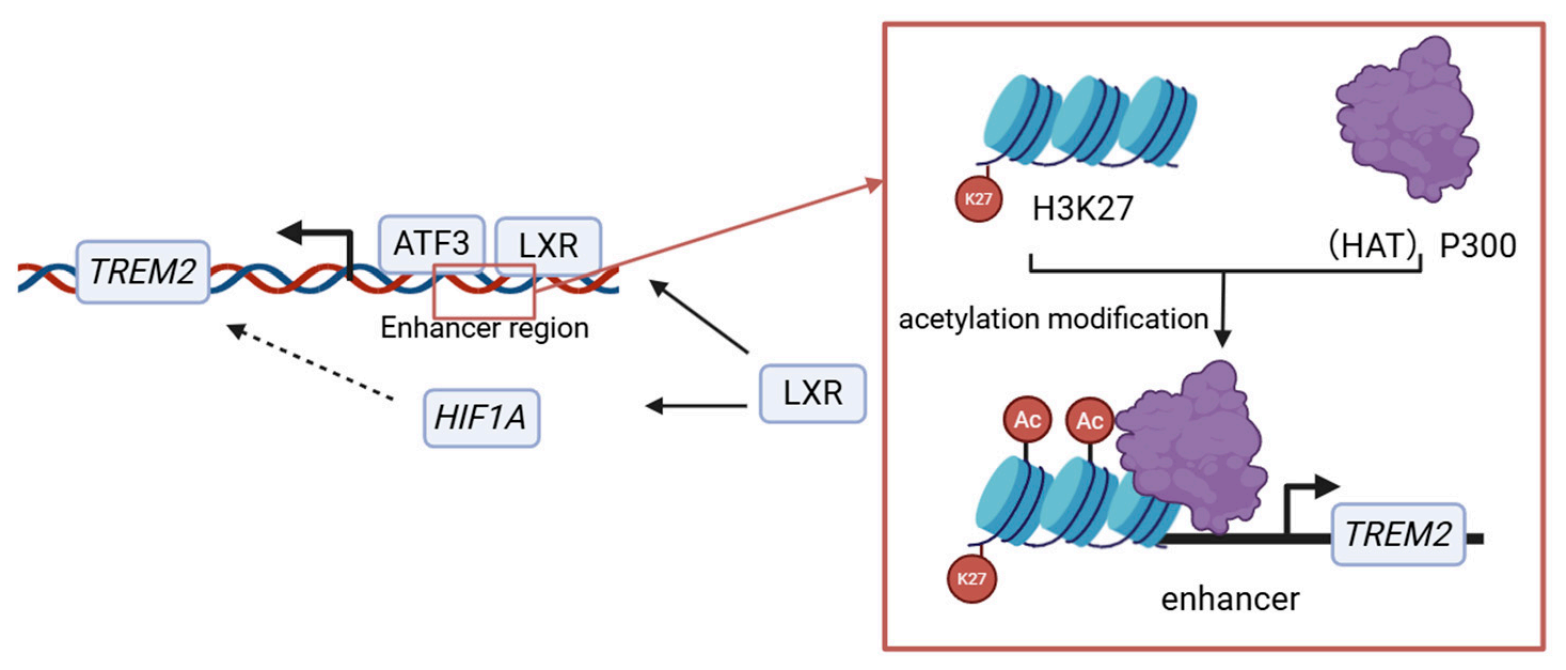
Liver X receptor(LXR) modulates TREM2 gene expression in macrophages. LXR and ATF3 co-bind to enhancer loci of genes including *Trem2* and *Cd9*, and subsequently recruit the histone acetyltransferase (HAT) p300. This complex catalyzes acetylation of histone H3 lysine 27 (H3K27), augments enhancer activity, and ultimately promotes *Trem2* expression. LXR also upregulates macrophage *TREM2* expression via hypoxia-inducible factor 1α *(HIF1A*). Solid arrows represent direct promotional effects, while dashed arrows represent indirect promotional effects.

**Figure 2:**
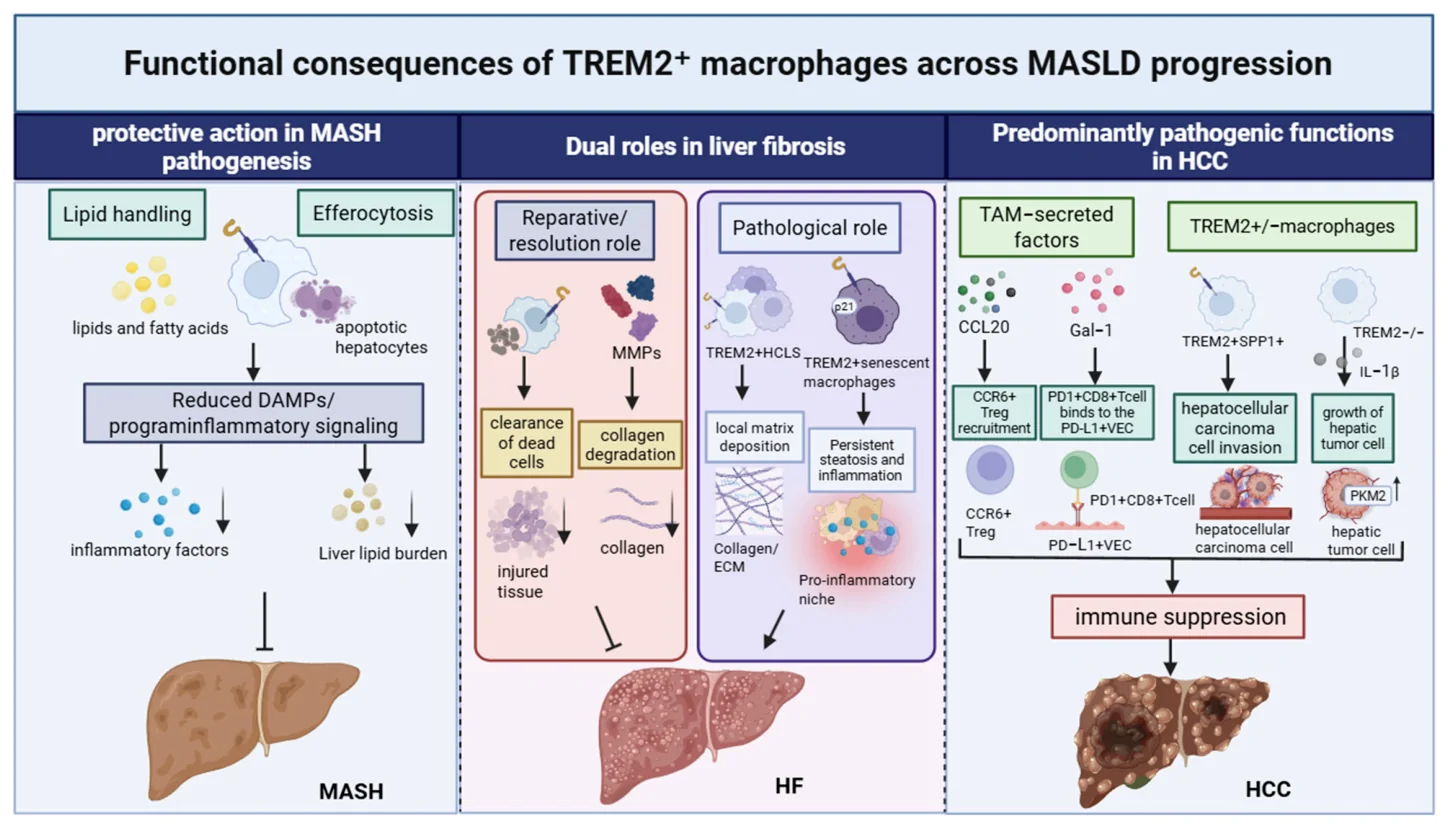
Functional consequences of TREM2^+^ macrophages across MASLD progression. During MASH, TREM2^+^ macrophages enhance intrahepatic fatty acid metabolism and phagocytose apoptotic hepatocytes, thereby reducing the release of damage-associated molecular patterns (DAMPs) and downstream proinflammatory signals. This may attenuate inflammatory cytokine production, hepatic lipid accumulation, and MASH progression. During hepatic fibrosis, TREM2^+^ macrophages exert opposing effects. On one hand, they facilitate the clearance of dead cells and tissue repair and may limit fibrogenesis through matrix metalloproteinase (MMP)-mediated collagen degradation. On the other hand, they may contribute to the formation of hepatic crown-like structures (HCLS), promote local extracellular matrix deposition, and maintain senescent p21^+^TREM2^+^ macrophage states that exacerbate hepatocellular steatosis and inflammation, thereby promoting fibrosis. During HCC progression, TREM2^+^ tumor-associated macrophages (TAMs) secrete CCL20 to recruit CCR6^+^ regulatory T cells (Tregs) to the liver and galectin-1 (Gal-1) to facilitate interactions between PD-1^+^ CD8^+^ T cells and PD-L1^+^ vascular endothelial cells (VECs), thereby restricting T-cell infiltration into the hepatic tumor microenvironment (TME). TREM2^+^SPP1^+^ macrophages may promote tumor-cell invasion, whereas TREM2-deficient macrophages release IL-1β, which enhances PKM2 activity and glycolysis in tumor cells, thereby promoting tumor growth. Together, these mechanisms contribute to the establishment of an immunosuppressive hepatic microenvironment and may accelerate HCC progression.

**Table 2:** Chemokine pathways involved in recruitment and positioning of TREM2^+^ macrophage precursors across MASLD-associated disease stages.

Chemokine	Receptor	Major Cellular Source/Context	Macrophage Population Affected	Evidence for TREM2 Induction	Disease Stage	References
CCL2 (MCP-1)	CCR2	Injured hepatocytes and other inflammatory hepatic cells	CCR2^+^ circulating monocytes/monocyte-derived macrophages	Ccr2 loss lowers hepatic Trem2 mainly by reducing precursor recruitment; direct regulation of Trem2 transcription has not been demonstrated	MASH; also HCC monocyte recruitment	[11,24]
CX3CL1	CX3CR1	Injured/stressed hepatic cells; niche-dependent	CX3CR1^+^ transitional monocyte-derived macrophages	Primarily affects migration, survival, and cell-state transition; direct Trem2 induction has not been established	MASH	[11,24,41]
CCL15	CCR1	HCC tumor/stromal context	Immunosuppressive recruited macrophages	Supports recruitment and localization of TREM2^+^ macrophages; not established as a direct Trem2 inducer	HCC	[42]
MDK	NCL	Malignant hepatocytes	TREM2^+^ macrophages	Directs recruitment and positioning rather than directly regulating Trem2 transcription	HCC	[43]

C-C motif chemokine ligand2 (CCL2), C-C motif chemokine receptor 2 (CCR2), C-X3-C motif chemokine ligand 1 (Cx3cl1), C-X3-C motif chemokine receptor 1 (Cx3cr1), C-C motif chemokine ligand15 (CCL15), C-C motif chemokine receptor 1 (CCR1), midkine (MDK), nucleolin (NCL).

## Data Availability

No new data were created or analyzed in this study. Data sharing is not applicable to this article.

## Abbreviations

adenosine 5′-monophosphate-activated protein kinase (AMPK), apolipoprotein A (APOA), apolipoprotein A/B/E (APOA/B/E), A disintegrin and metalloproteinase 10/17 (ADAM10/17), BCL2-associated X protein (BAX), C-C motif chemokine receptor 1 (CCR1), C-C motif chemokine receptor 2 (CCR2), C-C motif chemokine ligand 4 (CCL4), CCAAT/enhancer-binding protein α (CEBPα), cluster of differentiation 9 (CD9), cluster of differentiation 63 (CD63), C-X3-C motif chemokine ligand 1 (CX3CL1), C-X3-C motif chemokine receptor 1 (CX3CR1), carnitine palmitoyltransferase 1A (CPT1A), DNAX-activating protein 10 (DAP10), DNAX-activating protein 12 (DAP12), focal adhesion kinase (FAK), fatty acid binding protein 5 (FABP5), glucose transporter 1 (GLUT1), glycoprotein non-metastatic B (GPNMB), glycogen synthase kinase 3 beta (GSK3β), hexokinase 2 (HK2), hepatic crown-like structures (HCLS), hepatic stellate cells (HSCs), histone acetyltransferase (HAT), hypoxia-inducible factor 1α (HIF1A), interferon gamma (IFNγ), interleukin-1β (IL-1β), integrin subunit alpha V (ITGAV), intermediate-density lipoprotein (IDL), Kupffer cells (KCs), lipid droplets (LDs), lipid-associated macrophages (LAMs), lipoprotein lipase (LPL), low-density lipoprotein (LDL), matrix metalloproteinase 7 (MMP7), mitogen-activated protein kinase (MAPK), membrane-spanning 4-domains subfamily A member 7 (MS4A7), monocyte chemoattractant protein 1 (MCP1), monocyte-derived macrophages (MdMs), monocyte-derived Kupffer cells (Mo-KC), NASH-associated macrophages (NAMs), neural precursor cell expressed developmentally down-regulated 4-like (NEDD4L), neuregulin 4 (NRG4), nuclear factor-κB (NF-κB), NOD-like receptor family pyrin domain containing 3 (NLRP3), oxidized low-density lipoprotein (oxLDL), osteopontin (OPN), peroxisome proliferator-activated receptor (PPAR), platelet-derived growth factor subunit B (PDGF-B), platelet-derived growth factor receptor beta (PDGFRβ), programmed death-ligand 1 (PD-L1), pyruvate kinase M2 (PKM2), secreted phosphoprotein 1 (SPP1), scar-associated macrophages (SAMs), SMAD family member 3 (SMAD3), soluble triggering receptor expressed on myeloid cells 2 (sTREM2), sphingosine-1-phosphate (S1P), spleen tyrosine kinase (SYK), signal transducer and activator of transcription 3 (STAT3), toll-like receptor (TLR), transforming growth factor-beta-induced protein (TGFBI), triggering receptor expressed on myeloid cells 2 (TREM2), tumor necrosis factor α (TNFA), tumor necrosis factor superfamily member 12 (TNFSF12), tumor-associated macrophages (TAMs), unc-51 like autophagy activating kinase 1 (ULK1), vascular endothelial growth factor A (VEGFA), very-low-density lipoprotein (VLDL), tyrosine–isoleucine–asparagine–methionine motif (YINM), high-density lipoprotein (HDL), lipopolysaccharide (LPS), protein kinase B (AKT).
